# Analysis of the mitochondrial genome of the *Camellia sinensis* cv. ‘Zhuyeqi’: multichromosomal structure, RNA editing sites, and evolutionary characterization

**DOI:** 10.3389/fpls.2025.1644130

**Published:** 2025-10-02

**Authors:** Zhiyin Chen, Wei Zhou, Zixu Wang, Ziyi Chen, Xinyuan You, Yihui Gong

**Affiliations:** ^1^ College of Agriculture and Biotechnology, Hunan University of Humanities, Science and Technology, Loudi, Hunan, China; ^2^ Key Laboratory of Characteristic Agricultural Resource Development and Quality Safety Control in Hunan Province, Hunan University of Humanities, Science and Technology, Loudi, Hunan, China; ^3^ Innovation and Entrepreneurship Education Center for Horticultural Production and Processing in Hunan Province, Hunan University of Humanities, Science and Technology, Loudi, Hunan, China; ^4^ Hunan University of Humanities, Science and Technology, Hunan Provincial Innovation and Entrepreneurship Demonstration Base, Loudi, Hunan, China

**Keywords:** *Camellia sinensis*, mitochondrial genome, multichromosomal structure, RNA editing, phylogenetic relationships

## Abstract

**Introduction:**

Tea (*Camellia sinensis*) is a significant economic crop, and investigations into the structure and function of its mitochondrial genome are crucial for understanding the evolutionary history and genetic characteristics of this species. This study presents the first comprehensive analysis of the mitochondrial genome of the tea cultivar ’Zhuyeqi‘ (*Camellia sinensis* cv. ‘Zhuyeqi’), aiming to elucidate its genomic structural features, gene composition, and evolutionary patterns. The findings provide a theoretical foundation for genetic breeding and molecular biology research in tea plants.

**Methods:**

High-throughput sequencing was employed to sequence the mitochondrial genome of ’Zhuyeqi‘. Bioinformatics approaches were utilized for genome assembly and annotation. Various analytical strategies, including identification of RNA editing sites, codon usage bias analysis, repeat sequence recognition, calculation of non-synonymous substitution rates (Ka) and synonymous substitution rates (Ks), comparative genomics, and collinearity analysis, were applied to comprehensively analyze the structural features and evolutionary dynamics of the mitochondrial genome.

**Results and discussion:**

The mitochondrial genome of ‘Zhuyeqi’ consists of one circular chromosome and six linear chromosomes, with a total length of 911,255 bp and a GC content of 46%. Genome annotation identified 77 functional genes, including 38 protein-coding genes (PCGs). The study revealed heterogeneously distributed introns within genes such as trnM-CAT (5 copies) and *nad1/2/5/7*. RNA editing analysis identified 556 C-to-U editing sites, notably enriched in *ccmFn* (38 sites) and *ccmB* (34 sites). Codon usage bias analysis indicated that leucine (Leu, 10%) and arginine (Arg, 7%) were the most frequently used amino acids. Repeat sequence analysis showed that dispersed repeats (780, 72%) dominated, with satellite DNA exhibiting significant distribution biases on *chr1* (11) and *chr3* (5). Ka/Ks analysis revealed that 37 PCGs were under varying selective pressures (0.09–2.70), with *rps4* (Pi=0.09) and *atp8* (Pi=0.09) showing exceptionally high variability, while *rps19* (Pi=0) was completely conserved. Comparative genomics uncovered 66 homologous segments (25,656 bp) between the mitochondrial and chloroplast genomes, containing 27 intact genes such as *trnA-UGC*, confirming horizontal gene transfer events. Collinearity analysis demonstrated a high degree of conservation in genomic structures between ‘Zhuyeqi’ and closely related *Camellia* species. This study lays an important theoretical foundation for further elucidating the structural characteristics and evolutionary mechanisms of the tea plant mitochondrial genome.

## 1 Introduction

Mitochondria, as the central organelles of energy metabolism in eukaryotes, have genome dynamics and recombination mechanisms that are closely linked to plant environmental adaptation and evolution. Compared to the highly conserved chloroplast genome, plant mitochondrial genomes are typically characterized by multichromosomal configurations, frequent homologous recombination mediated by repeat sequences, and inter-organelle gene transfer. These structural complexities pose significant challenges to understanding species evolutionary trajectories (Li et al., 2023b, 2024). For example, the mitochondrial genome of Assam tea (*C. sinensis* var. assamica) contains a high proportion of repetitive sequences, reaching 43.9%, with a large number of quadruplicate repeats, which is significantly higher than the 37.2% found in Chinese small-leaf tea (*C. sinensis* var. sinensis) (Li et al., 2023b). Additionally, the mitochondrial genome size of Dahongpao (*C. sinensis* cv. Dahongpao) reaches 1,082,025 bp, which is significantly larger than the 991,788 bp of Rougui (*C. sinensis* cv. Rougui), suggesting that differences in chromosomal structure may be related to ecological adaptation (Li et al., 2024). Although studies on model plant mitochondrial genomes have made significant progress, the assembly of the tea tree (*Camellia sinensis*) mitochondrial genome still faces technical bottlenecks due to high heterogeneity and complex recombination events. In particular, there is a notable gap in research on the relationship between dynamic chromosomal configurations in cultivated varieties and ecological adaptability (Bi et al., 2022; Li et al., 2023b).

The *Camellia sinensis* cv. ‘Zhuyeqi’ is a core germplasm resource in China. Its chloroplast genome has been shown to possess a typical quadripartite structure, with no inversions or rearrangements in the inverted repeat (IR) regions and a unique localization of the *rps19* gene at the boundary between the large single-copy region and inverted repeat B (Chen et al., 2023). Notably, compared to the closely related species *Camellia japonica*, the IR region of *Camellia sinensis* cv. ‘Zhuyeqi’ has not undergone contraction. The distance of the *rpl2* gene from the LSC boundary is 106 bp, which is significantly different from the 112 bp in *C. sinensis* var. pubilimba (Chen et al., 2023). This suggests that the dynamics of genome boundaries may influence the efficiency of gene transfer between organelles. However, its mitochondrial genome lacks high-precision assembly data, and research is urgently needed to clarify its multichromosomal dynamic balancing mechanisms, functional network of ribonucleic acid (RNA) editing sites, and regulatory relationships with stress response pathways. Comparative studies show that 139 RNA editing sites were identified in the mitochondria of the Assam tea cultivar (*C. sinensis* var. *assamica* cv. Duntsa), with U-to-C editing accounting for 68%, which is significantly higher than the 52% in the small-leaf variety (*C. sinensis* var. sinensis) (Zhang et al., 2022). However, whether *Camellia sinensis* cv. ‘Zhuyeqi’ has a similar editing preference remains unknown. Notably, comparative genomic studies of closely related species indicate a significant positive correlation between mitochondrial genome heterogeneity and the degree of tea plant variety differentiation. For example, 40–46 chloroplast homologous fragments have been identified between the mitochondrial genomes of Assam tea (*C. sinensis* var. assamica) and Chinese small-leaf tea (*C. sinensis* var. sinensis), accounting for 1.91%-2.45% of the total mitochondrial length. Additionally, the *nad1* and *sdh3* genes show strong signals of positive selection in Assam tea (Li et al., 2024). The widespread phenomenon of multichromosomal coexistence in cultivated varieties may enhance ecological adaptability by regulating the activity of key enzymes in the tricarboxylic acid cycle (Zhao and Ma, 2021; Li et al., 2023b, 2024).

RNA editing, a key post-transcriptional regulatory mechanism in mitochondria, has identified 139 C-to-U editing sites in tea trees, with 32% of these events significantly altering the secondary structure of proteins. These include functional remodeling of key components of the electron transport chain, such as *nad4* and *cox1* (Zhang et al., 2022). It is noteworthy that, compared to the evergreen variety Longjing 43, the albino variety Huabai No. 1 shows a 90% reduction in intron splicing efficiency of the *ndhA* gene. Additionally, the editing level at the *matK-701* site in the yellowing variety is only 30% of that in the normal green-leaf variety (Gao, 2022; Zhang et al., 2022). Interestingly, abnormalities in chloroplast RNA editing have been significantly associated with tea tree albino phenotypes (e.g., the lack of editing at the *matK-701* site leads to chloroplast developmental defects) (Zhang et al., 2022), suggesting that mitochondrial editing may be involved in environmental adaptation by regulating reactive oxygen species metabolism. Adaptive evolution analysis shows that the *nad1* and *sdh3* genes have undergone strong positive selection in the tea plant lineage (Non-synonymous substitution rate (Ka)/Synonymous substitution rate (Ks) > 1). The non-synonymous mutation rate of the *sdh3* gene in the Assam variety of large-leaf tea (*C. sinensis* var. assamica) is 1.8 times higher than that in the Chinese small-leaf variety (*C. sinensis* var. sinensis), indicating significant differentiation in energy metabolism optimization strategies between the varieties (Li et al., 2024).Their non-synonymous mutation sites may enhance energy metabolism efficiency by optimizing the proton pump functions of complex I and II (Li et al., 2024). However, the cultivar-specific evolutionary trajectories (e.g., the 91 kb structural rearrangement in the mitochondrial genomes of CSSDP and CSSRG varieties (Li et al., 2024)) and their molecular associations with agronomic traits have yet to be systematically analyzed.

This study overcomes the traditional circular genome annotation paradigm by integrating short-read sequencing from Illumina NovaSeq 6000 and long-read sequencing from Nanopore PromethION (Wang et al., 2023). For the first time, it constructs a detailed multichromosomal map of the mitochondrial genome of the *Camellia sinensis* cv. ‘Zhuyeqi.’ Using comparative genomic methods, this study systematically analyzes the spatiotemporal expression patterns of RNA editing sites, combines positive selection site screening with population genetics data, and reveals the co-evolutionary mechanisms underlying multichromosomal dynamics and ecological adaptation. This research not only provides a new paradigm for the structural biology of the tea tree mitochondrial genome but also lays a molecular foundation for identifying targets for crop stress resistance breeding (Zhao and Ma, 2021; Bi et al., 2022; Li et al., 2023b).

## 2 Materials and methods

### 2.1 Preparation of mitochondrial genome DNA

**Figure 1:**
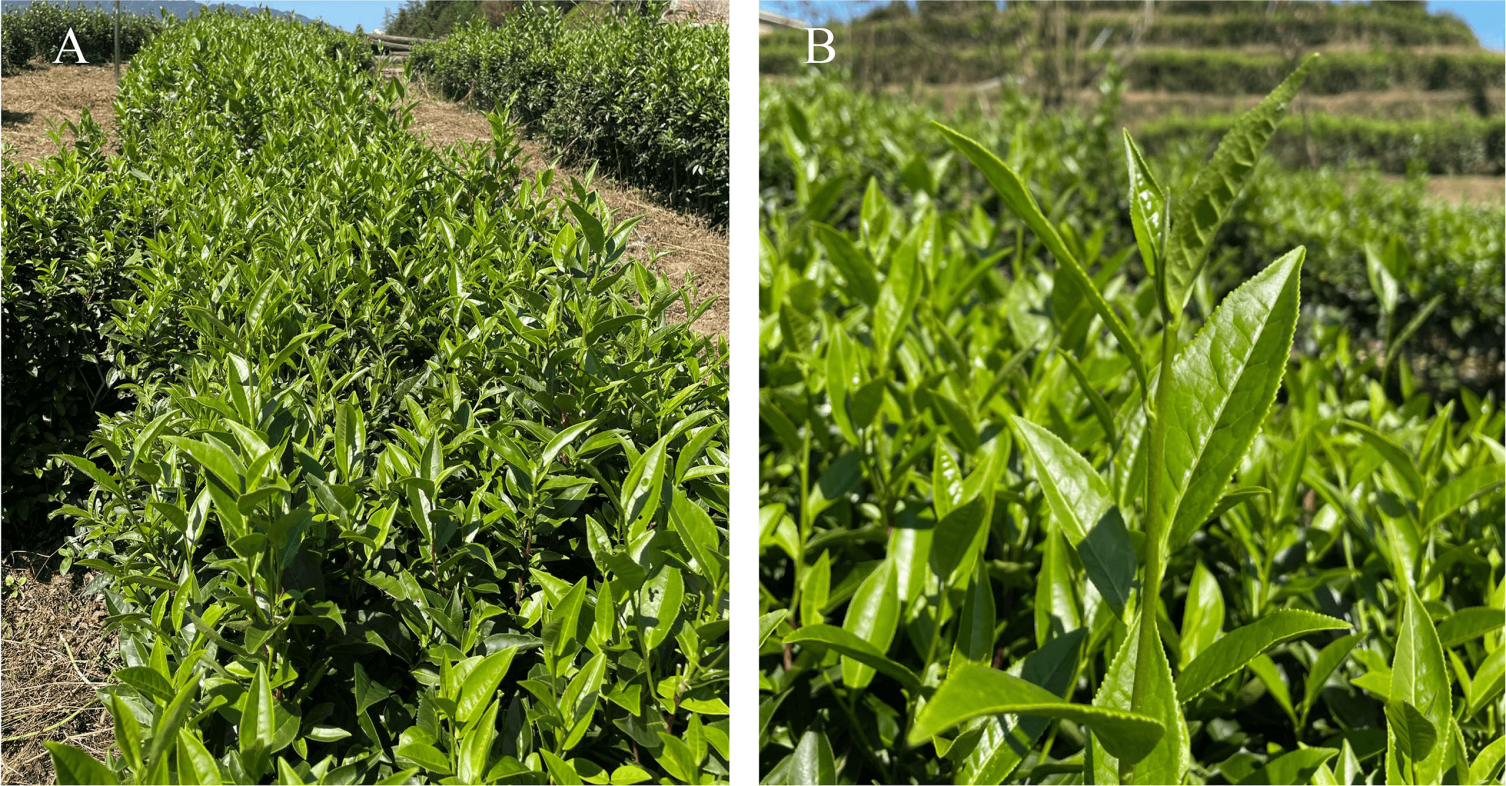
The planted field **(A)** and single tea plant of *Camellia sinensis* cv.’Zhuyeqi’ **(B)**.

In this study, young shoots with one bud and one leaf from the *Camellia sinensis* cv. ‘Zhuyeqi’, cultivated at the standardized tea planting demonstration base of Taohuayuan Agricultural Development Co., Ltd., Xinhua County, Hunan Province, China (27°47′N, 110°51′E), were used as experimental materials (**Figure 1**). Genomic deoxyribonucleic acid (DNA) was extracted using the TIANGEN Plant Genomic DNA Extraction Kit (DP305), following the manufacturer’s standard protocol. DNA purity was assessed using a NanoDrop spectrophotometer (Thermo Scientific), with A260/A280 ratios between 1.8 and 2.0 and A260/A230 ratios ≥2.0. DNA integrity was verified using 1% agarose gel electrophoresis (5 V/cm, 30 min), ensuring DNA fragment lengths >30 kb to meet the high molecular weight DNA requirements for long-read sequencing (Xu et al., 2018; Zhang et al., 2019; Chen et al., 2023).

### 2.2 Sequencing data generation and quality control

A combined strategy using second-generation (Illumina NovaSeq 6000) and third-generation (Oxford Nanopore PromethION) sequencing technologies was employed to balance read coverage and long-fragment assembly accuracy (Zhang et al., 2019; Wang et al., 2023). Second-generation sequencing library preparation: Genomic DNA was fragmented into 300–500 bp fragments using a Covaris system, followed by end repair, 3’ poly-A tailing, and Illumina adapter ligation. The library quality was assessed using a Qubit 3.0 fluorometer and an Agilent 2100 Bioanalyzer. Paired-end sequencing (2×150 bp) was performed on the NovaSeq 6000 platform (Zhang et al., 2019). Raw sequencing data were filtered using *fastp* (v0.23.4) to remove low-quality reads based on the following criteria:1) Reads containing adapter or primer sequences. 2) Reads with an average Phred quality score<Q5. 3) Reads with an N base proportion >5% (An et al., 2021; Li et al., 2024). Third-generation sequencing and quality control: DNA fragments >10 kb were enriched using a magnetic bead-based selection method, followed by end repair and adapter ligation using the SQK-LSK109 library preparation kit. Third-generation sequencing data were filtered using *filtlong* (v0.2.1) with parameters: –min_length 1000 and –min_mean_q 7. Perl scripts were used to analyze the read N50, average quality scores, and guanine-cytosine (GC) content to ensure the data met the requirements for high-accuracy assembly (Li et al., 2023b; Wang et al., 2023).

### 2.3 Mitochondrial genome assembly

A hierarchical iterative assembly framework was constructed based on the high conservation of core plant mitochondrial genes (e.g., *nad5*, *atp9*, *cox2*) (Li et al., 2024). Third-generation sequencing data (PacBio/Nanopore) were initially aligned using *minimap2* (v2.1). High-confidence seed sequences were selected based on alignment lengths >50 bp and coverage of at least three core genes to avoid interference from repetitive sequences (Li et al., 2024). Seed sequences were extended through multiple rounds of iterative alignment (overlap >1 kb) and integrated into an initial assembly dataset. To address the unique subcircular and non-circular topologies of tea tree mitochondria (Zhang et al., 2019; Bi et al., 2022; Li et al., 2024), third-generation sequencing data were corrected using *Canu*, and a hybrid assembly strategy was implemented by combining second-generation data (mapped using Bowtie2 v2.3.5.1) with *Unicycler* (v0.4.8). This approach significantly improved haplotype coverage depth and assembly continuity (Wang et al., 2024b). Further analysis using the visualization tool *Bandage* (v0.8.1) was performed to examine contig branching patterns. Manual corrections were applied to refine multichromosomal structure boundaries, based on differences in coverage depth and the distribution of repeat sequences. This process enabled the precise reconstruction of the complex multichromosomal configuration (Bi et al., 2022; Li et al., 2024). This strategy effectively overcame the fragmentation issues caused by high repeat sequences, providing a solid structural foundation for subsequent adaptive evolution analyses (Li et al., 2024; Wang et al., 2024b).

### 2.4 Functional annotation of mitochondrial genes

A multidimensional annotation strategy was applied: (1) Core gene annotation: The mitochondrial genomes of closely related species were used as a reference database. Homologous sequences were identified using *BLAST* (E-value<1e-5). Exon-intron boundaries were optimized using *Geneious Prime*, and chloroplast homologous fragments were manually excluded (threshold: sequence similarity ≥80% (Lu et al., 2022; Li et al., 2023b). (2) Transfer RNA (tRNA) identification: tRNAs were predicted using *tRNAscan-SE* (v2.0) under the default eukaryotic mode, with secondary validation by *ARWEN* to ensure stability of tRNA secondary structures (Zhang et al., 2019; Li et al., 2024). (3) Open reading frame (ORF) screening and functional annotation: Non-redundant sequences ≥102 bp were identified using *ORF Finder* (National Center for Biotechnology Information, NCBI). Overlapping regions with known genes and low-complexity regions (analyzed with *RepeatMasker*) were excluded. ORFs ≥300 bp were annotated using *BLASTX* against the nr database, focusing on gene clusters related to adaptive evolution Complete chloroplast genome sequence of Camellia sinensis genome structure, adaptive evolution, and phylogenetic relationships (Li et al., 2024).

### 2.5 Functional analysis of RNA editing sites

To systematically analyze the functional characteristics of mitochondrial RNA editing sites in the *Camellia sinensis* cv. ‘Zhuyeqi,’ this study employed an integrated multi-omics strategy. First, RNA-seq data generated from the Illumina platform were aligned to mitochondrial genome coding regions using *Bowtie2* (v2.3.5.1) with high precision. The alignment results in BAM format were then sorted and filtered for quality using *SAMtools* (v1.9) (Li et al., 2009; Danecek et al., 2021). Next, single nucleotide polymorphism (SNP) sites between genomic DNA and transcriptome data were identified using *BCFtools* (v1.9-170). These results were integrated with predictions from the Predictive RNA Editor for Plant Mitochondria to construct a high-confidence set of RNA editing sites (Mower, 2009; Danecek et al., 2021). To further validate the biological significance of editing events, the Kyte-Doolittle hydropathy index was used to quantify changes in the physicochemical properties of amino acids induced by editing (Zhang et al., 2022). Finally, a generalized linear model was applied to analyze the association between RNA editing sites and signals of positive selection (Ka/Ks >1), revealing the regulatory role of RNA editing in the adaptive evolution of tea tree mitochondria (Zhang et al., 2022; Li et al., 2023b).

### 2.6 Relative synonymous codon usage analysis

To investigate the codon usage bias and the evolutionary driving forces in the mitochondrial genome of the tea plant variety ‘Zhuyeqi’, this study used the Relative Synonymous Codon Usage (RSCU) index for quantitative analysis. The RSCU is calculated using Equation 1:


(1) $$RSCU=\frac{f_{obs}}{f_{exp}}$$

Here, $f_{obs}$ represents the observed frequency of a synonymous codon, and $f_{exp}\;\;$ represents the expected frequency under a neutral evolutionary model. An RSCU value of 1 indicates neutral selection, while RSCU > 1 or< 1 suggests positive or negative selection pressure, respectively (Zhang et al., 2022; Li et al., 2023b). To eliminate redundancy from homologous sequences, unique mitochondrial coding sequences were filtered using CD-HIT (identity = 100%). The RSCU values were then calculated and statistically tested in batches using a custom Perl script, available at https://github.com/ari-dasci/S-TSFE-DL. The expected frequencies were based on a uniform distribution model (Fu et al., 2012).

### 2.7 Analysis of repetitive sequences in the mitochondrial genome

To systematically analyze the characteristics of repetitive sequences in the mitochondrial genome, this study utilized a multi-dimensional strategy for repetitive sequence detection: (1) Simple Sequence Repeats (SSR): Whole-genome scanning for SSRs was performed using the *MISA* software (v1.0). The parameters were set based on plant mitochondrial genome research standards. The minimum repeat numbers for mono- to hexanucleotide repeat units were set as 10, 5, 4, 3, 3, and 3, respectively, to balance detection sensitivity and specificity (Sahu et al., 2012; Zhang et al., 2019). (2) Tandem Repeats: Tandem repeats were analyzed using the *TRF algorithm* (v4.0.9). The parameters were set as follows: match score = 2, mismatch penalty = 7, indel penalty = 7, and minimum alignment score = 80. This ensured the effective identification of periodic repeat units ≥50 bp in length (Benson, 1999; Wynn and Christensen, 2019). (3) Dispersed Repeats: Dispersed repeats were detected by performing a whole-genome self-comparison using *BLASTn* (v2.10.1), with optimized settings for word size (word_size = 7) and significance threshold (evalue = 1e-5). The REPuter algorithm was then used to remove redundant signals and to exclude tandem repeat interference (Wynn and Christensen, 2019; Liu et al., 2024). Finally, a multi-level interaction network map of repetitive sequences was constructed using *Circos* (v0.69-5), revealing the spatial distribution patterns of repetitive elements within the genome (Zhang et al., 2019).

### 2.8 Adaptive evolution (Ka/Ks) analysis

Based on the homologous gene clusters of the following reference species: *Zea mays* subsp. *mays* (NC_007982.1), *Brassica oleracea* (NC_016118.1), *Gossypium barbadense* (NC_028254.1), *Gossypium arboreum* (NC_035073.1), *Styphnolobium japonicum* (NC_039596.1), *Chrysanthemum boreale* (NC_039757.1), and *Camellia sinensis* (NC_043914.1), an evolutionary pressure model was constructed. First, global multiple sequence alignment was performed using *MAFFT* v7.427 (Katoh and Standley, 2013), and low-conserved regions were removed with Gblocks. Then, Ka and Ks values were calculated using the MLWL algorithm in KaKs_Calculator v2.0 (Li et al., 2024), with the significance threshold set at *p*< 0.05. Particular focus was given to genes under positive selection (Ka/Ks > 1) and genes under strong purifying selection (Ka/Ks< 0.5), which were further analyzed for functional associations with previously reported core functional genes in tea plant mitochondria (Li et al., 2024). Boxplots of Ka/Ks values across evolutionary branches were constructed using the ggpubr package in R, and an evolutionary model of selection pressure over time and across lineages was integrated with PhyloTree (Wang et al., 2024b).

### 2.9 Nucleotide diversity (Pi Value) analysis

Based on the MAFFT multiple sequence alignment (parameters: –retree 1 –maxiterate 1000) of homologous gene clusters from the following reference species: *Zea mays* subsp. *mays* (NC_007982.1), *Brassica oleracea* (NC_016118.1), *Gossypium barbadense* (NC_028254.1), *Gossypium arboreum* (NC_035073.1), *Styphnolobium japonicum* (NC_039596.1), *Chrysanthemum boreale* (NC_039757.1), and *Camellia sinensis* (NC_043914.1), nucleotide polymorphism analysis was performed using the sliding window method (window length: 500 bp, step size: 100 bp) (Li et al., 2024). The average Pi value of single nucleotide polymorphism sites was calculated using *DnaSP* v5.0, and confidence intervals were assessed through 1,000 bootstrap resampling iterations to quantify the level of genetic differentiation among species (Li et al., 2024; Wang et al., 2024b). To eliminate interference from low-quality alignment regions, highly variable sites were filtered prior to alignment using Gblocks v0.91b (allowing gaps with the parameter: -b5=h) (Wang et al., 2024b).

### 2.10 Phylogenetic tree construction

Based on the multiple sequence alignment of whole-genome coding sequences generated by MAFFT, conserved regions were selected using *trimAl* v1.4 (parameters: -gt 0.7 -cons 60) (Li et al., 2024) to retain high-confidence sites. The optimal evolutionary model was determined using *PartitionFinder* v2.1.1, supporting the GTR+GAMMA+I model (Lanfear et al., 2016). A maximum likelihood tree was constructed with *RAxML* v8.2.10 (parameters: -m GTRGAMMAI -f a -x 12345), and Bayesian inference was performed using *MrBayes* v3.2.6 (number of chains = 4, sampling frequency = 1,000, convergence criterion: average standard deviation< 0.01). Finally, 1,000 bootstrap support values and posterior probabilities were integrated to enhance the reliability of the tree topology (Li et al., 2024; Wang et al., 2024b).

### 2.11 Mitochondrial genome synteny and plastid homologous sequence analysis

Whole-genome synteny alignment was performed using *nucmer* v4.0 (parameters: –maxmatch -c 65 -l 40), and syntenic blocks and rearrangement events were identified with *SyRI* v1.6 (Li et al., 2023b). High-resolution dot plots were generated using *ggplot2* (Wickham, 2017). For mitochondrial-to-chloroplast horizontal transfer sequences, BLASTN (threshold: E-value ≤ 1e-10, coverage ≥ 80%) (Altschul et al., 1997) was combined with *GeneWise* v2.4.1 to verify open reading frame integrity (Zhang et al., 2019) and exclude false-positive transfer events. Cross-genome interaction networks were visualized with *CIRCOS* v0.69-5, and the dynamics of genome structure were revealed based on SSRs and long terminal repeats identified by *REPuter* v2.74 (Kurtz et al., 2001; Li et al., 2023b; Liu et al., 2024).

## 3 Results

### 3.1 Multichromosomal structure of the mitochondrial genome in *Camellia sinensis* cv. ‘Zhuyeqi’

**Figure 2:**
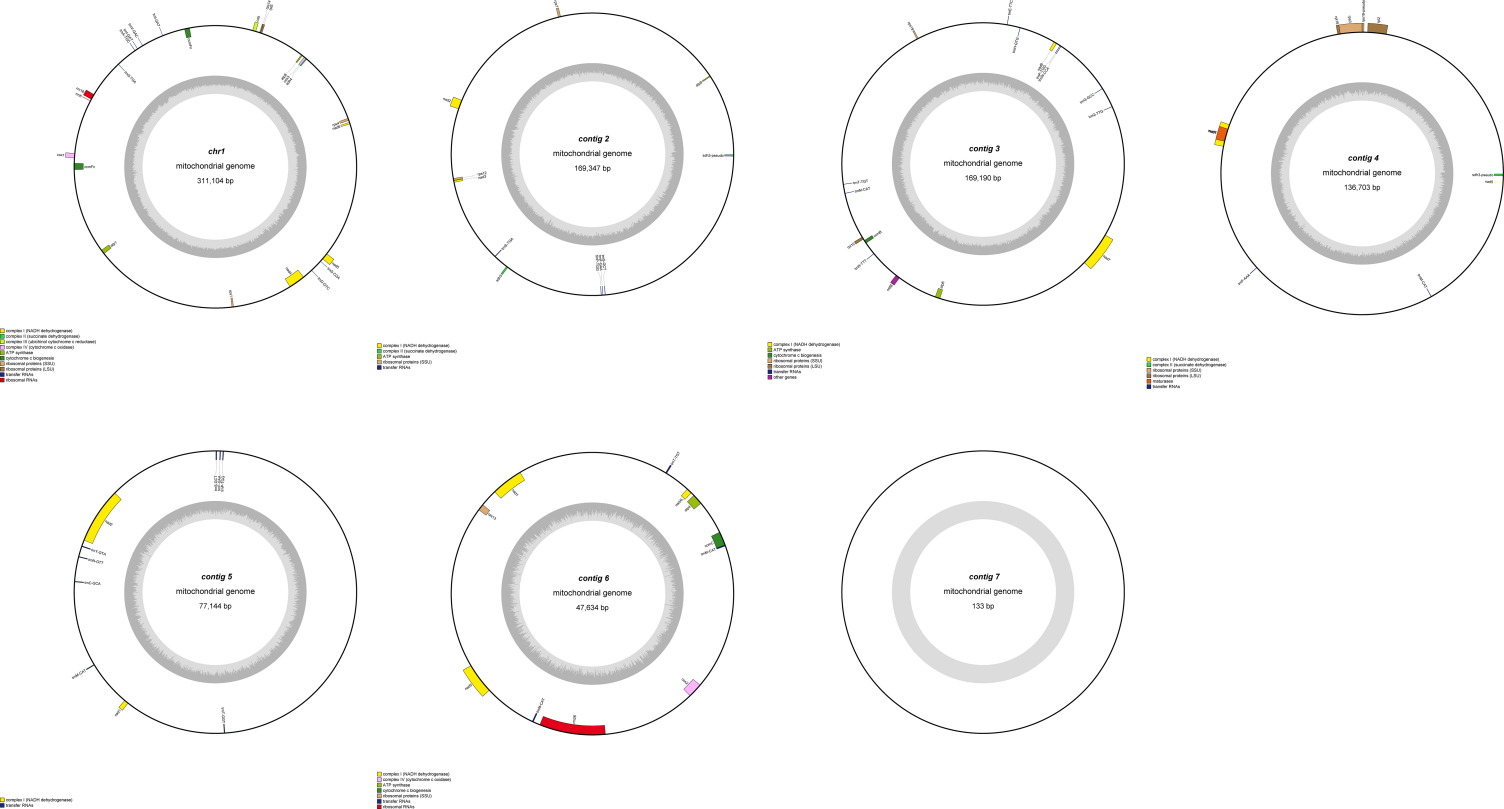
Mitochondrial genome map of *Camellia sinensis* cv. ‘Zhuyeqi’. The mitochondrial genome consists of seven chromosomes. Genes encoded on the forward strand are located on the outer side of the circle, while genes encoded on the reverse strand are on the inner side. The inner gray circle represents GC content.

This study is the first to elucidate the complex multichromosomal configuration of the mitochondrial genome in the *Camellia sinensis* cv. ‘Zhuyeqi’ (GenBank accession numbers: PQ763484–PQ763490). The genome exhibits a unique structure composed of one circular chromosome and six linear chromosomes (**Figure 2**; **Supplementary Table 1**), with a total length of 911,255 bp (individual chromosome lengths ranging from 133 to 311,104 bp) and a GC content of 46% (**Supplementary Table 2**). The genome size and GC content are highly conserved compared to previously reported *Camellia* species (701,719–1,081,966 bp, GC 44–46%), supporting the evolutionary stability of mitochondrial genomes in the Theaceae family (Li et al., 2023b). Gene annotation identified 77 functional genes, including 38 protein-coding genes (PCGs), 33 tRNAs, 3 ribosomal RNAs (rRNAs), and 3 pseudogenes (**Table 1**; **Supplementary Table 3**). The core functional modules include adenosine triphosphate (ATP) synthase (*atp1/4/6/8/9*), nicotinamide adenine dinucleotide (NADH) dehydrogenase (*nad1–7/9*), and cytochrome c oxidase (*cox1–3*), consistent with the conserved characteristics of mitochondrial genomes in terrestrial plants (Lu et al., 2022; Chen et al., 2023). Notably, the GC content of PCGs (43%) is significantly lower than that of tRNAs (50%) and rRNAs (48%) (**Supplementary Table 2**). This nucleotide composition heterogeneity may be associated with functional differentiation or post-transcriptional regulatory mechanisms (Chen et al., 2023; Li et al., 2024).

**Table 1:** Functional classification of genes in the mitochondrial genome of *Camellia sinensis* cv. ‘Zhuyeqi’.

Group of genes	Gene name	Length	Start codon	Stop codon	Amino acid
ATP synthase	*atp1*	1530	ATG	TGA	510
	*atp4*	579	ATG	TAA	193
	*atp6*	948	ATG	TAG	316
	*atp8*	480	ATG	TAA	160
	*atp9*	225	ATG	CGA(TGA)	75
Cytohrome c biogenesis	*ccmB*	621	ATG	TGA	207
	*ccmC*	753	ATG	TGA	251
	ccmFc*	1317	ATG	CGA(TGA)	439
	*ccmFn*	1797	ATG	TAG	599
Ubichinol cytochrome c reductase	*cob*	1182	ATG	TGA	394
Cytochrome c oxidase	*cox1*	1584	ACG(ATG)	TAA	528
	*cox2*	792	ATG	TAA	264
	*cox3*	798	ATG	TGA	266
Maturases	*matR*	1968	ATG	TAG	656
Transport membrance protein	*mttB*	837	ATG	TAG	279
NADH dehydrogenase	nad1****	978	ATG	TAA	326
	nad2****	1467	ATG	TAA	489
	*nad3*	357	ATG	TAA	119
	nad4**	1488	ATG	TGA	496
	*nad4L*	303	ACG(ATG)	TAA	101
	nad5****	2013	ATG	TAA	671
	*nad6*	618	ATG	TAA	206
	nad7****	1185	ATG	TAG	395
	*nad9*	573	ATG	TAA	191
Ribosomal proteins	*rpl10*	489	ATG	TAA	163
	*rpl16*	516	ATG	TAA	172
	rpl2*	1014	ATG	TAA	338
	*rpl5*	564	ATG	TAA	188
Ribosomal proteins	#*rps19*(2) rps1*	285 513	ATG ATG	TAA TAA	95 171
	*rps12*	378	ATG	TGA	126
	*rps13*	351	ATG	TGA	117
	*rps14*	303	ATG	TAG	101
	rps3*	1692	ATG	TAG	564
	*rps4*	831	ATG	TAA	277
	*rps7*	447	ATG	TAA	149
Succinate dehydrogenase	#*sdh3*(2)	321	ATG	TGA	107
	*Sdh3* *sdh4*	321 387	ATG ATG	TGA CGA(TGA)	107 129
Ribosomal RNAs	*rrn18*	25859			
	*rrn26*	3599			
	*rrn5*	121			
Transfer RNAs	*trnA*-TGC*	65			
	*trnC*-GCA	71			
	*trnD*-GTC	74			
	*trnE*-TTC	72			
	*trnF*-AAA*	69			
	*trnF*-GAA(2)	74			
	*trnG*-GCC	72			
	*trnH*-GTG	74			
	*trnI*-GAT*(2)	68			
	*trnK*-TTT	73			
	*trnM*-CAT(5)	74			
	*trnN*-GTT	72			
	*trnP*-TGG(3)	75			
	*trnQ*-TTG	72			
	*trnS*-CGA	58			
	*trnS*-GCT(2)	88			
	*trnS*-TGA	87			
	*trnS*-TGA*	77			
	*trnT*-GGT*	69			
	*trnT*-TGT*(2)	69			
	*trnV*-GAC	72			
	*trnW*-CCA	74			
	*trnY*-GTA	83			
other					

*indicates one intron, **indicates two introns, ****indicates four introns. (2) indicates two copies, (3) indicates three copies, (4) indicates four copies, # indicates pseudogenes.

Structural analysis of the genome revealed multiple copy numbers for some genes, such as *trnM-CAT* (5 copies), *trnP-TGG* (3 copies), and *trnF-GAA*, *trnI-GAT*, and four other genes with two copies each. These duplications suggest that recombination events mediated by repeat sequences or horizontal gene transfer might drive genome structural variation (Li et al., 2024; Liu et al., 2024). Additionally, the presence of the *sdh3* pseudogene may reflect functional redundancy and differentiation of the mitochondrial genome during evolution, aligning with the adaptive evolutionary strategies of the *Camellia* genus (Li et al., 2023b, 2024). Moreover, the distribution of introns exhibits high heterogeneity: nine genes, including *ccmFc* and *rpl2*, contain a single intron; *nad4* contains two introns, while *nad1/2/5/7* each contain four introns. This complex splicing pattern may contribute to the fine regulation of gene expression through alternative splicing, closely related to the dynamic plasticity of the mitochondrial transcriptome in tea plants (Zhang et al., 2019, 2022). Similar intron structures have also been reported in *Camellia sinensis* var. *assamica*, suggesting that these may represent conserved regulatory features of mitochondrial genomes in the Theaceae family (Zhang et al., 2019).

### 3.2 Prediction and analysis of RNA editing sites

**Figure 3:**
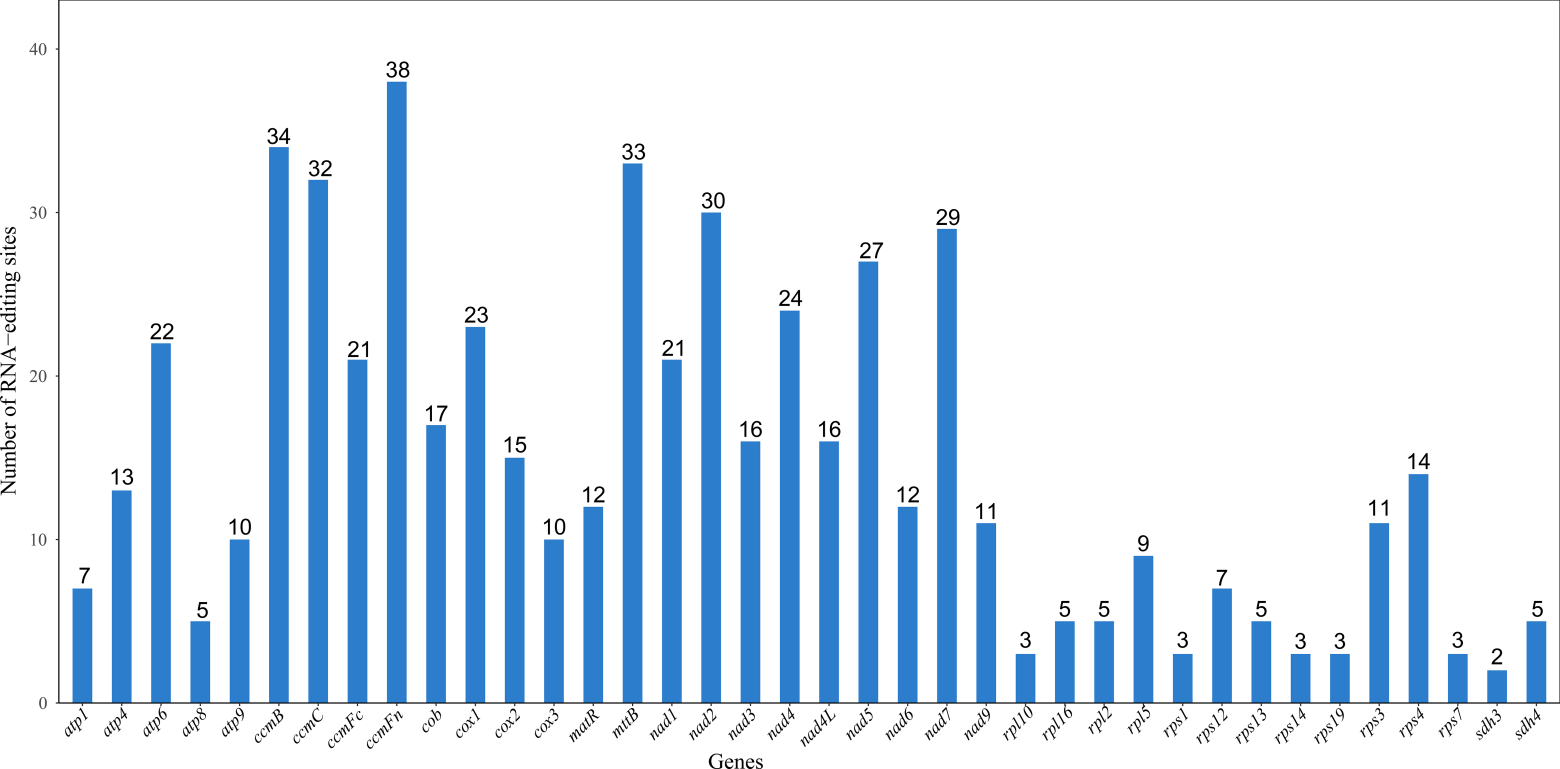
Statistics of RNA editing sites for each gene. The horizontal axis represents genes, and the vertical axis represents the number of RNA editing sites.

This study systematically analyzed 38 PCGs in the mitochondrial genome of the *Camellia sinensis* cv. ‘Zhuyeqi’ and identified a total of 556 RNA editing sites (**Figure 3**), with C-to-U editing being the predominant type. This result is consistent with the dominant C-to-U editing pattern observed in mitochondrial genomes of angiosperms (Li et al., 2021; Zhang et al., 2022). The editing sites were unevenly distributed among the genes: the *ccmFn* gene contained the most editing sites (38), followed by *ccmB* (34), *mttB* (33), and *nad2* (30), while *sdh3* had only 2 sites. Notably, the high density of editing sites in the *ccmB* gene (34 sites) aligns with reports from other tea cultivars (Zhang et al., 2022), and its features under positive selection may reflect its critical role in mitochondrial energy metabolism and its adaptive evolutionary demands (Li et al., 2024; Zhang et al., 2024). Additionally, the high number of editing sites in *ccmFn* is similar to the editing hotspots observed in mitochondrial genes of plants like Populus, suggesting that strong selective pressure due to functional conservation may act on this gene during cross-species evolution (Zhang et al., 2022, 2024).

The amino acid substitutions caused by RNA editing were classified into five categories: hydrophilic-to-hydrophilic, hydrophilic-to-hydrophobic, hydrophilic-to-stop codon, hydrophobic-to-hydrophilic, and hydrophobic-to-hydrophobic (**Table 2**). Among these, hydrophilic-to-hydrophobic substitutions were the most frequent (48%), with typical examples including TCA (S, serine) → TTA (L, leucine), TCG (S) → TTG (L), and CGG (R, arginine) → TGG (W, tryptophan). These substitutions significantly enhanced the local hydrophobicity of proteins, which may contribute to remodeling transmembrane domains or the hydrophobic cores of enzyme active sites, thereby regulating the assembly efficiency and functionality of mitochondrial oxidative phosphorylation complexes (Li et al., 2021; Zhang et al., 2022). Hydrophobic-to-hydrophobic substitutions accounted for 31% (e.g., CCA → CTA causing P → L, GCC → GTC causing A → V). Although these substitutions did not change amino acid polarity, differences in side-chain volume and hydrophobicity may lead to subtle conformational adjustments, such as altering the packing density of ATP synthase transmembrane helices or the spatial constraints of substrate-binding pockets (Zhang et al., 2019; Duan and Lai, 2020). Hydrophilic-to-stop codon substitutions were the least frequent (1%), such as CAG (Q) → TAG (X) and CGA (R) → TGA (X).

**Table 2:** Changes in amino acid hydropathy properties caused by RNA editing.

Type	RNA-editing	Number	Percentage
hydrophilic-hydrophilic	CAC (H) => TAC (Y)	9	
	CAT (H) => TAT (Y)	21	
	CGC (R) => TGC (C)	13	
	CGT (R) => TGT (C)	32	
	total	75	13%
hydrophilic-hydrophobic	ACA (T) => ATA (I)	4	
	ACC (T) => ATC (I)	1	
	ACG (T) => ATG (M)	7	
	ACT (T) => ATT (I)	4	
	CGG (R) => TGG (W)	34	
	TCA (S) => TTA (L)	79	
	TCC (S) => TTC (F)	35	
	TCG (S) => TTG (L)	52	
	TCT (S) => TTT (F)	50	
	total	266	48%
hydrophilic-stop	CAG (Q) => TAG (X)	1	
	CGA (R) => TGA (X)	3	
	total	4	1%
hydrophobic-hydrophilic	CCA (P) => TCA (S)	8	
	CCC (P) => TCC (S)	9	
	CCG (P) => TCG (S)	5	
	CCT (P) => TCT (S)	19	
	total	41	7%
hydrophobic-hydrophobic	CCA (P) => CTA (L)	51	
	CCC (P) => CTC (L)	8	
	CCC (P) => TTC (F)	8	
	CCG (P) => CTG (L)	38	
	CCT (P) => CTT (L)	21	
	CCT (P) => TTT (F)	14	
	CTC (L) => TTC (F)	6	
	CTT (L) => TTT (F)	14	
	GCC (A) => GTC (V)	1	
	GCG (A) => GTG (V)	7	
	GCT (A) => GTT (V)	2	
	total	170	31%
	All	556	100%

Type, Type of hydropathy change; RNA-editing, RNA editing type; Number, Number of RNA editing events; Percentage, Proportion of total events.

### 3.3 RSCU analysis

**Figure 4:**
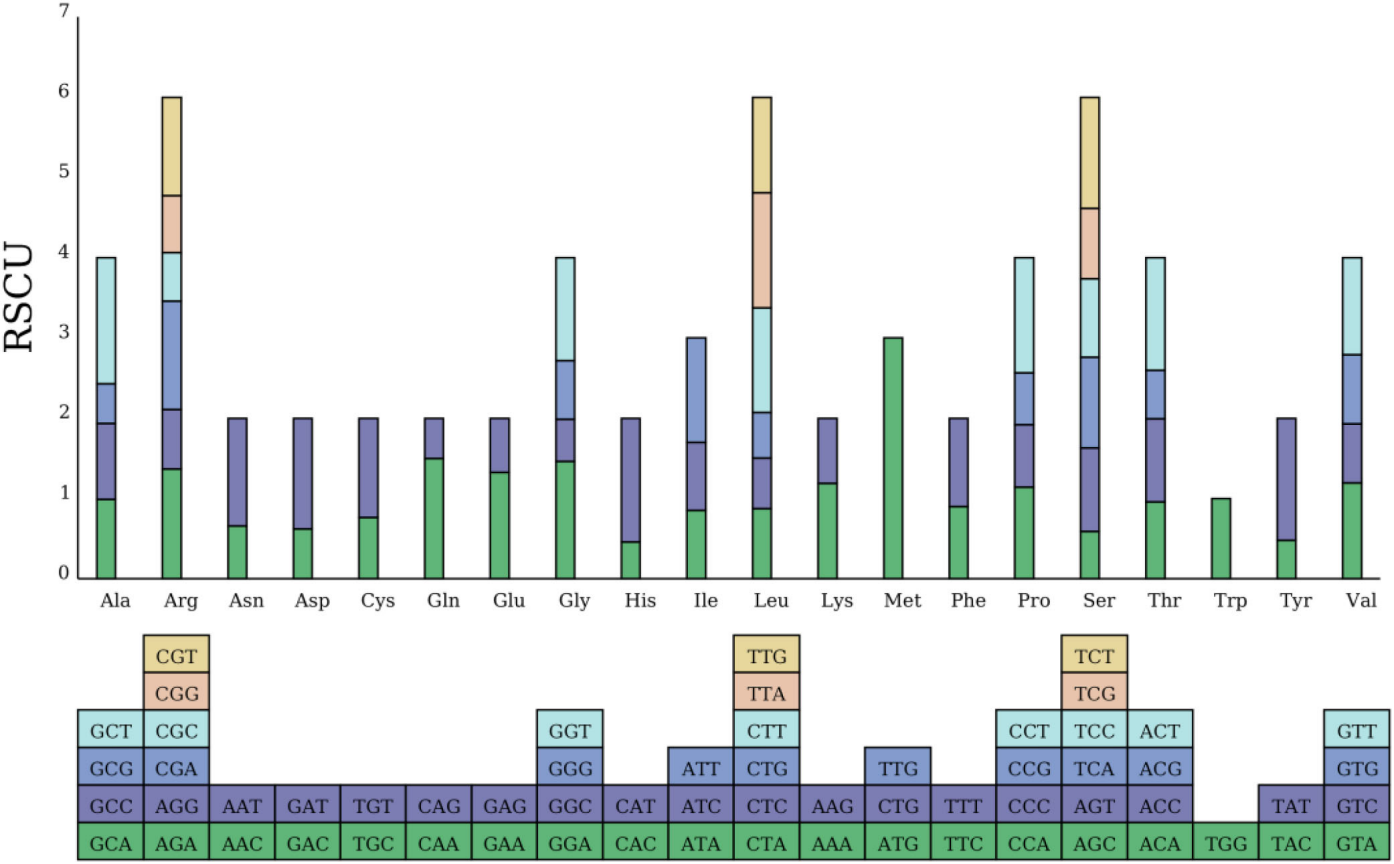
RSCU in the *Camellia sinensis* cv.’Zhuyeqi’mitogenome. The blocks below represent all codons encoding each amino acid. The height of the columns above represents the total RSCU values of all codons.

This study conducted a codon usage analysis on 10,828 codons from 38 PCGs in the mitochondrial genome of the *Camellia sinensis* cv. ‘Zhuyeqi’. The results revealed a significant codon usage bias pattern. Among aliphatic amino acids, leucine (Leu) had the highest expression frequency (1,113 occurrences, accounting for 10%), followed by serine (Ser, 9%) and arginine (Arg, 7%), whereas tryptophan (Trp) showed a significantly lower usage frequency (1%) compared to other amino acids (**Figure 4**; **Supplementary Table 4**). This distribution pattern is highly consistent with the conserved evolutionary trends of mitochondrial genomes in *Camellia* species (Mebeaselassie and Zhu, 2022; Li et al., 2024). Notably, among aromatic amino acids, the phenylalanine (Phe) codon UUU exhibited extreme preference (379 occurrences, 4%), with a 23% higher usage frequency than its synonymous codon UUC (3%). This observation aligns with the preferential selection of A/T-ending codons in the chloroplast genomes of Theaceae plants (Wang et al., 2022). Similarly, the isoleucine (Ile) codon AUU (3%) and the glutamic acid (Glu) codon GAA (3%) also displayed high expression frequencies. In contrast, no expression activity was detected for the methionine (Met) codons CUG/UUG, suggesting a potential functional deficit in the mitochondrial translation system (Mebeaselassie and Zhu, 2022; Chen et al., 2023).

Across the genome, 31 codons with relative synonymous codon usage (RSCU) values >1 were identified, accounting for 47% of all codons. Among these, the methionine start codon AUG had the highest RSCU value (3.0) in the genome. This result is consistent with the conserved functional characteristics of start codons reported by Li et al. (2024) in a comparative mitochondrial genomics study of tea variants (Li et al., 2024). Analysis of effective number of codons (ENC) and GC3s data (**Supplementary Table 5**) showed that high-RSCU codons were predominantly found in AU-rich regions with low GC content (e.g., AUU, UUU). This is highly related to the adaptive evolutionary strategy of terrestrial plant mitochondrial genomes, which reduce translation energy costs through codon deoptimization (Wang et al., 2022; Li et al., 2024).

### 3.4 Multidimensional analysis of repetitive sequence features

**Figure 5:**
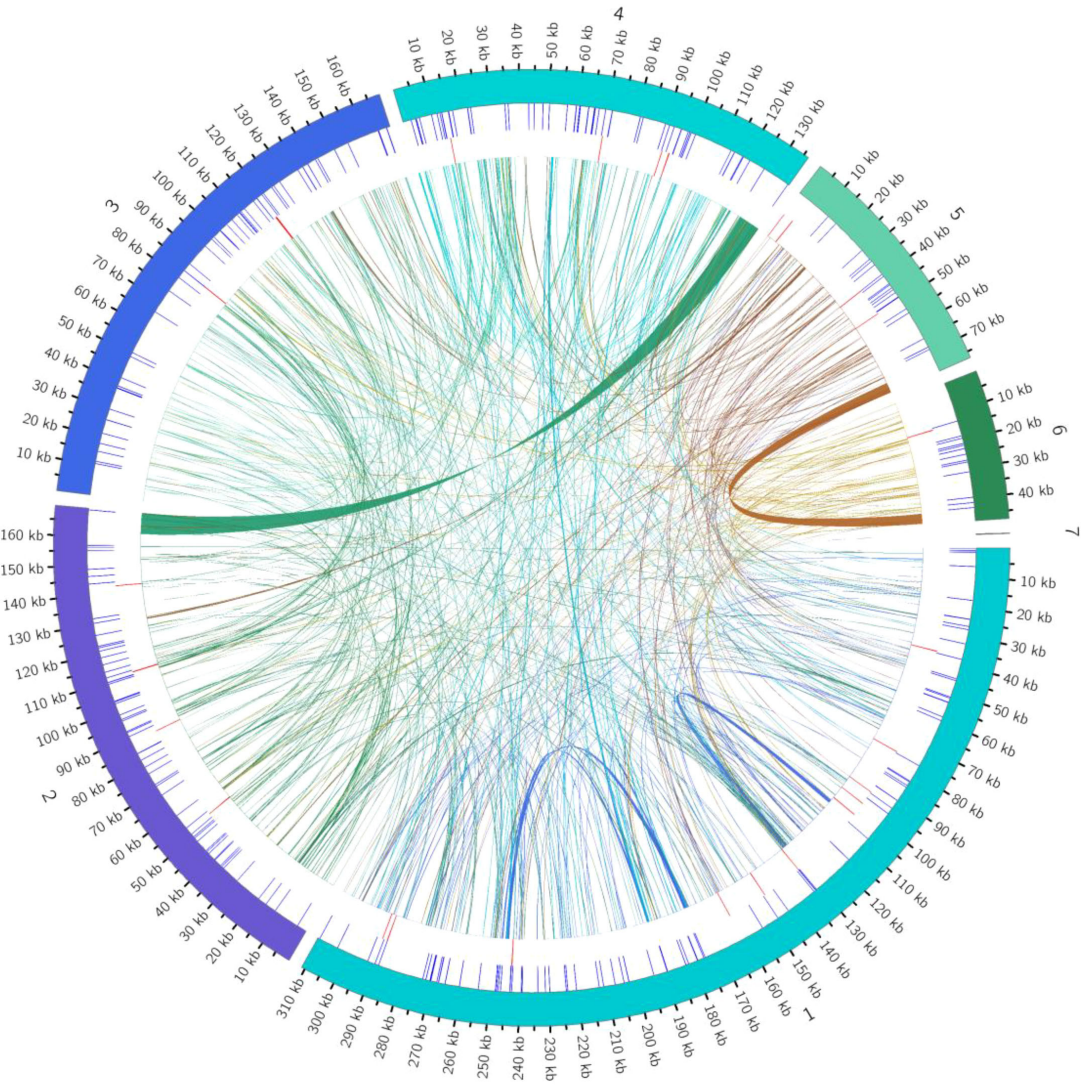
Distribution of repetitive sequences in the genome. The outermost circle represents the mitochondrial genome sequence. Moving inward, the circles represent simple sequence repeats, tandem repeats, and dispersed repeats, respectively.

The mitochondrial genome of the *Camellia sinensis* cv. ‘Zhuyeqi’ exhibits a typical multilayered repetitive sequence structure, including SSRs, tandem repeats, and dispersed repeats. Quantitative analysis identified a total of 1,078 repetitive sequences, with SSRs (269, 25%) and tandem repeats (29, 3%) as minor components, while dispersed repeats (780, 72%) were the predominant type (**Figure 5**; **Supplementary Table 6**). This distribution pattern aligns with the conserved characteristics of tea mitochondrial genomes, suggesting that dispersed repeats play a central role in driving the dynamic evolution of the mitochondrial genome (Li et al., 2024).

The total length of dispersed repeats was 68,976 bp, accounting for 8% of the genome, which is significantly higher than reported for other tea cultivars (e.g., *C. sinensis* var. *assamica*) (Huang et al., 2014; Li et al., 2023b). The distribution showed marked heterogeneity among chromosomes, with *chr4* (8,082 bp), *chr6* (3,900 bp), and *chr1* (1,281/1,277 bp) having the largest alignment lengths. This may be associated with localized recombination suppression or active transposon activity (Huang et al., 2014). Subtype analysis revealed that forward repeats (354, 45%) and palindromic repeats (426, 55%) were the major types (**Supplementary Table 7**). Notably, *chr4* contained the largest forward repeat (8,082 bp), while *chr1* carried the largest palindromic repeat (1,281 bp). These findings support the hypothesis that mitochondrial genomes maintain structural stability through homologous recombination (Zhang et al., 2019; Li et al., 2023b). Short dispersed repeats (<100 bp) accounted for 86% (668) of all dispersed repeats. Their high abundance suggests that DNA slippage replication may drive genome microevolution, a phenomenon widely observed in the adaptive evolution of plant mitochondrial genomes (Li et al., 2023a, 2024).

Satellite DNA analysis identified 29 tandem repeat units with lengths ranging from 6–39 bp, showing significant asymmetry in chromosome distribution: *chr1* was enriched with 11 sites, *chr3* had 5, *chr6* only 1, and *chr7–11* lacked tandem repeats entirely (**Supplementary Table 8**). This uneven distribution is associated with the compartmentalized multichromosomal structure of mitochondria and may reflect the regulation of mitochondrial genome 3D conformation by nuclear-mitochondrial interactions (Huang et al., 2014; Liu et al., 2024).

### 3.5 Ka/Ks analysis

**Figure 6:**
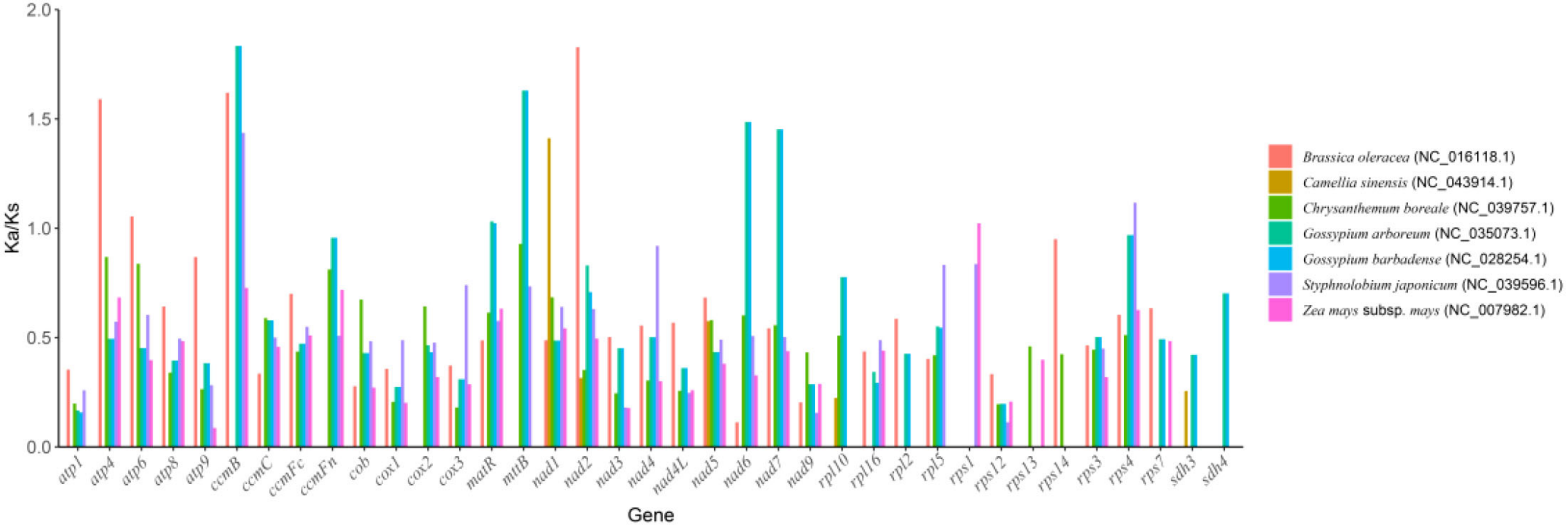
Boxplot of Ka/Ks values.

In molecular evolution studies, the ratio of Ka to Ks is a key indicator for assessing the selective pressure on protein-coding genes. This study systematically compared the evolutionary dynamics of 37 core functional genes in the mitochondrial genome of the *Camellia sinensis* cv.’Zhuyeqi’with those of representative species, including *Camellia sinensis* (NC_043914.1), *Brassica oleracea* (NC_016118.1), *Chrysanthemum boreale* (NC_039757.1), *Gossypium arboreum* (NC_035073.1), *G. barbadense* (NC_028254.1), *Styphnolobium japonicum* (NC_039596.1), and *Zea mays subsp. mays* (NC_007982.1) (**Figure 6**; **Supplementary Table 9**). The analysis revealed cross-lineage adaptive evolutionary characteristics.

The Ka/Ks values for the 37 PCGs ranged from 0.09 to 2.70 (**Supplementary Table 10**), consistent with the Ka/Ks distribution patterns observed in the mitochondrial genome of *Camellia* species [2]. Notably, the *atp9* gene of *Zea mays subsp. mays* showed the lowest Ka/Ks value (0.09), indicating strong purifying selection, likely associated with the functional conservation of the ATP synthase complex in energy metabolism (Xia et al., 2014; Huang et al., 2022).

In contrast, the *sdh4* gene of *Chrysanthemum boreale* exhibited the highest Ka/Ks value (2.70), followed by the *ccmB* gene (2.22), suggesting that these genes may have undergone positive selection. This finding aligns with the positive selection observed for *Nad1* and *Sdh3* genes in the mitochondrial genome of *Camellia* species, highlighting the convergent evolution of key metabolic genes (e.g., succinate dehydrogenase complex and cytochrome c maturation-related genes) in plant adaptive evolution (Li et al., 2024). Notably, the average Ka/Ks value for the *ccmB* gene was 1.38, significantly exceeding the neutral evolution threshold (Ka/Ks = 1). Its positive selection characteristics appear conserved across multiple plant lineages (Li et al., 2023b, 2024).

### 3.6 Nucleotide diversity analysis

**Figure 7:**
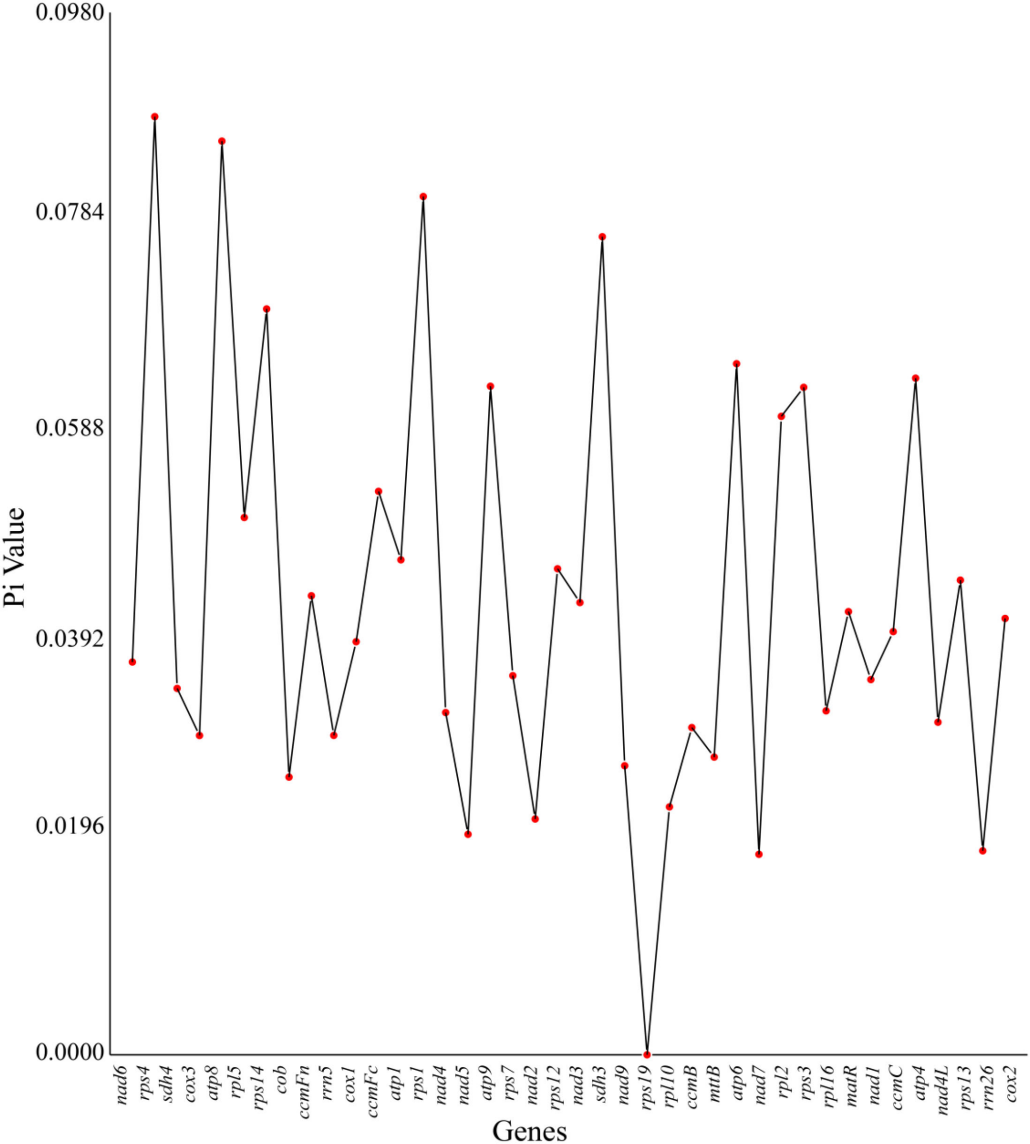
Nucleotide diversity in the mitochondrial genome of *Camellia sinensis* cv. ‘Zhuyeqi’.

This study conducted a phylogenetic analysis of nucleotide diversity (Pi) for 40 core functional genes in the mitochondrial genome of the *Camellia sinensis* cv. ‘Zhuyeqi’ (**Figure 7**; **Supplementary Table 11**). The results revealed multidimensional heterogeneity in genetic variation. The Pi values of functional genes showed a wide range (0–0.09), with the ribosomal protein genes *rps4* (Pi = 0.09), ATP synthase subunit gene *atp8* (0.09), and *rps1* (0.08) exhibiting exceptionally high levels of variation. In contrast, *rps19* (Pi = 0) was completely conserved, suggesting it is under strong functional constraint or purifying selection (Ka/Ks< 1) (Li et al., 2024). Notably, the succinate dehydrogenase gene *sdh3* (Pi > 0.07) and *rps14* (Pi > 0.07) displayed high levels of variation, which is consistent with previous studies reporting positive selection (Ka/Ks > 1) in the *nad1* and *sdh3* genes (Li et al., 2024), further supporting the critical role of mitochondrial genomes in the adaptive evolution of tea plants (Li et al., 2024).

Among rRNA genes, *rrn5* (Pi = 0.03) showed significantly higher variation compared to *rrn26* (Pi = 0.02), while the absence of Pi values for *rrn18* may be attributed to its highly conserved secondary structure and RNA editing mechanisms. Studies have shown that mitochondrial RNA editing is predominantly C-to-U, with a strong preference for T bases in the surrounding sequences (Li et al., 2024). This editing pattern likely contributes to maintaining functional stability by regulating translation efficiency (Zhang et al., 2019). Comparative analysis of genomic structures revealed significant differences in variation patterns between mitochondrial and chloroplast genomes. The chloroplast genome exhibited stronger conservation in inverted repeats (IRs) (Pi< 0.005) (Peng et al., 2021), whereas the mitochondrial genome showed increased structural plasticity due to frequent recombination and horizontal gene transfer events (Li et al., 2024).

### 3.7 Homologous sequence analysis between mitochondrial and chloroplast genomes

**Figure 8:**
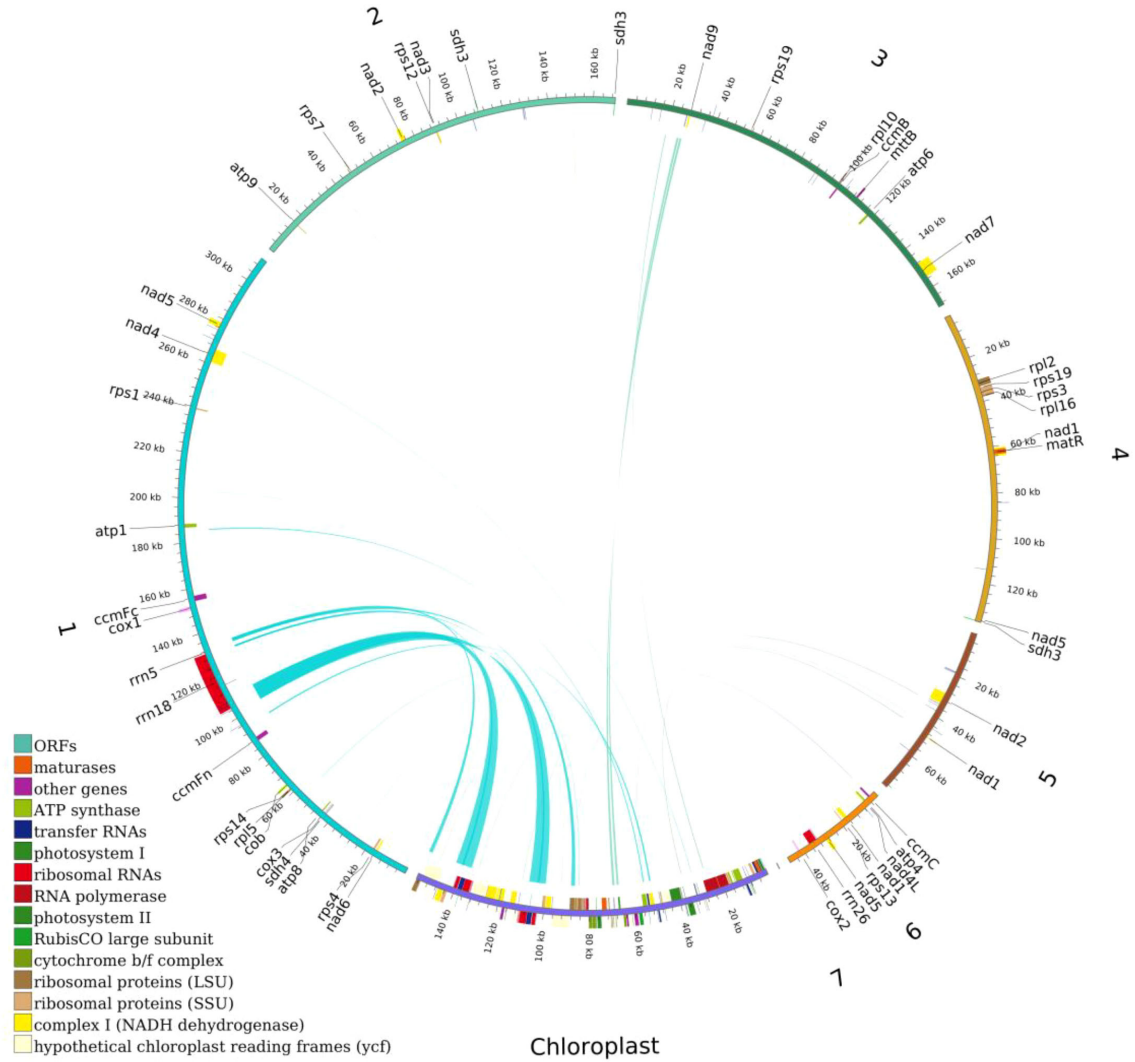
Distribution of homologous sequences between the mitochondrial and chloroplast genomes of *Camellia sinensis* cv. ‘Zhuyeqi’. “Chloroplast” represents chloroplast sequences, while other labels represent mitochondrial sequences. Genes from the same complex are represented by blocks of the same color. Blocks on the outer and inner circles indicate genes on the positive and negative strands, respectively. The connecting lines in the middle indicate homologous sequences.

This study systematically analyzed the homologous sequences between the mitochondrial and chloroplast genomes of the *Camellia sinensis* cv. ‘Zhuyeqi’. The mitochondrial genome, with a total length of 911,255 bp, is approximately 5.8 times larger than the chloroplast genome (157,072 bp) (**Figure 8**; **Supplementary Table 12**), consistent with the reported size variation of tea mitochondrial genomes (701,719–1,081,966 bp) (Li et al., 2023b). Whole-genome alignment identified 66 homologous fragments between the chloroplast and mitochondrial genomes, ranging from 32 to 7,721 bp, with a total length of 25,656 bp (**Supplementary Table 13**). These homologous sequences account for 15% of the chloroplast genome and 2% of the mitochondrial genome. The proportion of mitochondrial homologous sequences (2%) falls within the typical range (1.91%-2.45%) reported for other tea cultivars (Li et al., 2024). This proportion differs significantly from legumes, such as *Dalbergia odorifera*, where chloroplast-derived sequences account for approximately 4% of the mitochondrial genome (Hong et al., 2021), but both systems highlight the widespread occurrence of inter-organellar DNA transfer (Wang et al., 2012; Li et al., 2023b). It is important to note that the evolutionary rates and recombination mechanisms of plant mitochondrial and chloroplast genomes differ significantly (Sloan et al., 2012; Tyszka et al., 2023), and no direct quantitative relationship has been established between the proportion of homologous sequences and the activity of DNA transfer (Li et al., 2024; Wang et al., 2024a). Therefore, the observed proportion of homologous sequences may represent a baseline level of gene flow between organelles in tea plants, while the activity of the specific transfer mechanisms requires further analysis, including recombination frequency and insertion site characteristics (Hong et al., 2021; Li et al., 2024).

In the chloroplast genome, homologous sequences (23,747 bp, 15%) contain 27 complete genes, including key functional genes such as *trnA-UGC*, *rrn16*, and *rpl2*. Additionally, the homologous regions of *rps12*, *atpE*, and *rrn16* covered 75%, 91%, and 58% of their original gene lengths, respectively (**Supplementary Table 13**). This suggests that chloroplast DNA transferred to the mitochondria may preferentially retain functional domains (Li et al., 2023b). In contrast, the mitochondrial homologous sequences (14,776 bp, 2%) included only 14 complete genes (e.g., *trnV-GAC* and *trnW-CCA*), with some homologous genes showing coverage below 20% (**Supplementary Table 13**). This indicates that the mitochondrial genome may impose stricter selection or degradation mechanisms on transferred sequences (Li et al., 2024). Such differences may be driven by the strong purifying selection acting on the mitochondrial genome (Li et al., 2024).

### 3.8 Phylogenetic and evolutionary analysis

**Figure 9:**
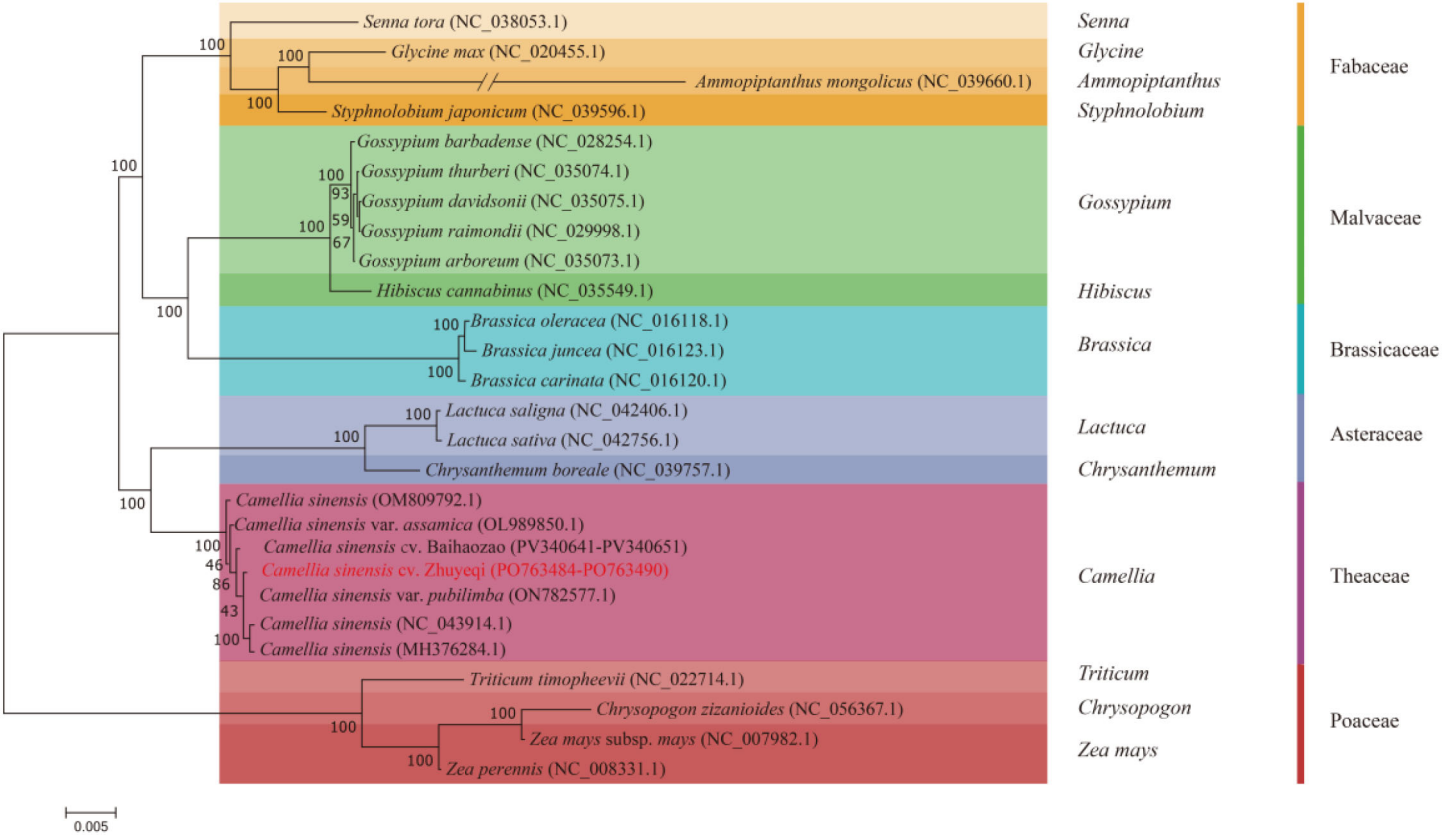
Phylogenetic relationships between *Camellia sinensis* cv. ‘Zhuyeqi’ and 25 other plant species.

A maximum likelihood phylogenetic tree constructed based on 38 mitochondrial protein-coding genes (**Figure 9**) revealed five monophyletic groups among 26 representative species. The study selected three *Poaceae* species, *Triticum timopheevii* (NC_022714.1), *Chrysopogon zizanioides* (NC_0656367.1), and *Zea mays* (NC_007982.1), as outgroups (Yang et al., 2024a). The remaining species clustered into branches corresponding to Theaceae, Asteraceae, Brassicaceae, Malvaceae, and Fabaceae. Notably, the *Camellia sinensis* cv. ‘Zhuyeqi’ clustered wit1h 100% bootstrap support within the Camellia branch, forming a monophyletic group with *C. sinensis* (OM809792.1) and the Assam variety *C. sinensis* var. *assamica* (OL989850.1). This result aligns with the monophyly of the Camellia tribe observed in phylogenetic analyses based on the *matR* gene (Yang et al., 2006). Genetic differentiation distance analysis showed that *Camellia sinensis* cv. ‘Zhuyeqi’ shares a closer relationship with three major cultivated tea varieties—*C. sinensis* var. *pubilimba* (ON782577.1), *C. sinensis* (NC_043914.1), and *C. sinensis* (MH376284.1)—than the inter-family differentiation threshold. This finding suggests that these varieties may have originated from a shared ancestral population that underwent rapid radiation evolution (Xu, 2019; Li et al., 2024).

### 3.9 Synteny analysis of the mitochondrial genome

**Figure 10:**
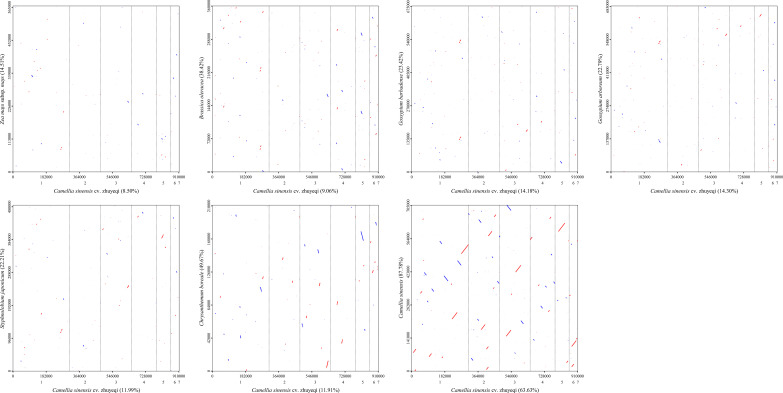
Homologous sequence alignment between the mitochondrial genome of *Camellia sinensis* cv. ‘Zhuyeqi’ and seven other species. The horizontal axis in each box represents the assembled sequence, while the vertical axis represents sequences from other species. The values in parentheses indicate the proportion of homologous sequences in the total genome. Red lines in the boxes represent forward alignments, while blue lines indicate reverse complementary alignments.

This study conducted a synteny analysis of the mitochondrial genome of the *Camellia sinensis* cv. ‘Zhuyeqi’ using whole-genome alignment strategies (**Figure 10**; **Supplementary Table 14**). The results revealed significant structural conservation between *Camellia sinensis* cv. ‘Zhuyeqi’ and other *Camellia sinensis* species, with homologous regions covering 64% (579,821 bp) of the *Camellia sinensis* cv. ‘Zhuyeqi’ genome and 88% (620,962 bp) of the other Camellia genome. This observation contrasts sharply with the frequent gene rearrangements and heterogeneous repeat sequences commonly found in Camellia mitochondrial genomes (Lu et al., 2022; Li et al., 2024). Notably, the structural conservation in the *Camellia sinensis* cv. ‘Zhuyeqi’ may be attributed to artificial selection pressures during domestication, which preserved core functional modules. This aligns closely with the “domestication bottleneck effect” theory proposed by Li et al. (2024) (Li et al., 2024). Specifically, although the mitochondrial genome of *Camellia* species can contain up to 44% SSRs and exhibit numerous re6combination hotspots (Li et al., 2023b), the observed conservation in this study may result from the selective stabilization of key functional units such as respiratory chain complexes (Wei et al., 2018). Cross-species comparisons showed that syntenic regions between *Camellia sinensis* cv. ‘Zhuyeqi’ and the monocot *Zea mays* subsp. *mays* accounted for only 9% (77,430 bp) of the genome. This difference is likely due to genome remodeling events triggered by early divergences among angiosperms (Wei et al., 2018; Yang et al., 2024a).

## 4 Discussion

This study, for the first time, reveals that the mitochondrial genome of the tea plant variety ‘Zhuyeqi’ exhibits a “1 circular + 6 linear” composite chromosome configuration (with a total length of 911,255 bp), which forms a significant contrast to the typical quadripartite circular structure of the ‘Zhuyeqi’ chloroplast. The chloroplast genome of ‘Zhuyeqi’ has a total length of 157,072 bp, encoding 52 protein-coding genes (CDS), 8 rRNA genes, and 37 tRNA genes, and maintains genomic stability through inverted repeat (IR) regions (Zeng et al., 2025). In contrast, the mitochondrial genome relies on dispersed repeat sequences (accounting for 72%) to mediate chromosome breakage/fusion events. The mitochondrial genome of ‘Baihaozao’ consists of 11 linear chromosomes (with a total length of 909,843 bp and a GC content of 45.62%). This structural difference leads to significantly higher recombination activity in the mitochondria (with forward repeats accounting for 45%) compared to the chloroplast (SSR density of only 5.3/kb) (Chen et al., 2025).This structural dynamic balance may be maintained by homologous recombination mediated by direct repeats (45%) and palindromic repeats (55%), similar to the recombination mechanisms of the multi-chromosomal mitochondrial structures in vascular plants such as *Quercus acutissima*, which rely on intramolecular recombination mediated by repetitive sequences to maintain genome plasticity (Wynn and Christensen, 2019; Liu et al., 2022). Notably, the modular recombination characteristics of linear chromosomes may optimize mitochondrial translation efficiency through the dosage effects of multi-copy genes such as *trnM-CAT* (5 copies) and *trnP-TGG* (3 copies). This optimization likely supports the metabolic differentiation between young shoots and mature leaves of tea plants. Compared to the conserved intron distribution in the mitochondrial genome of sweet tea (*Rosa rubus*) from the Rosaceae family (4 introns in *nad1/2/5/7*), the dynamic loss of introns in *nad4* (only 2 introns) in *Camellia sinensis* cv. ‘Zhuyeqi’ reveals a unique intron degradation pathway in the *Camellia* genus (Chen et al., 2023; Li et al., 2024).

A total of 556 C→U RNA editing sites were identified in the mitochondrial PCGs of *Camellia sinensis* cv. ‘Zhuyeqi’, with the highest editing densities observed in *ccmFn* (38 sites) and *nad2* (30 sites). This indicates precise regulation of the assembly of electron transport chain complexes (Zhang et al., 2022). Comparative analysis shows that the number of editing sites in *Camellia sinensis* cv. ‘Zhuyeqi’ is significantly higher than the 478 sites in large-leaf tea (*Camellia sinensis* var. assamica) but lower than the 635 sites in *Camellia gigantocarpa*, suggesting a positive correlation between RNA editing intensity and mitochondrial genome complexity within the *Camellia* genus (Zhang et al., 2019; Lu et al., 2022). While the editing pattern is conserved with *C. nitidissima* and *C. taliensis*, the number of editing sites is significantly higher than that in *C. taliensis* (492 sites), suggesting functional divergence in RNA editing among tea species (Li et al., 2024). The editing event at position 701 of the chloroplast *matK* gene in ‘Huabai No. 1’ is directly associated with leaf color variation, while the C→U conversion at position 15 among the 30 editing sites of the mitochondrial *nad2* gene significantly affects the activity of the respiratory chain complex (Yang et al., 2024b). Notably, the downregulation of the CsMORF9.2 factor in ‘Baiye No. 1’ leads to reduced RNA editing efficiency across multiple organelles, highlighting the synergistic role of editing factors between organelles (Yang et al., 2024b).

The high usage frequency of aliphatic amino acids (Leu/Ser/Arg, 27%) corresponds to the high nitrogen metabolism characteristics of tea plants, which may improve the hydrophobicity of respiratory chain proteins and enhance electron transport chain efficiency (Li et al., 2023b; Wang et al., 2024b). This feature is conserved in closely related species of the *Camellia* genus. For example, the mitochondrial genome of *Camellia gigantocarpa* shows a leucine (Leu) content as high as 18.5%, significantly higher than that of *Arabidopsis thaliana* (12.3%) and Asteraceae plants (14.1%), suggesting a unique nitrogen metabolism adaptation strategy in the *Camellia* genus (Lu et al., 2022). The extreme preference for the phenylalanine codon UUU (4%) may regulate the biosynthesis of phenylpropanoids, a feature also observed in the chloroplast genome of *C. taliensis* (Chen et al., 2023). The high-frequency usage of aliphatic amino acids (Leu/Ser/Arg accounting for 27%) in the mitochondrial genome complements the A/U-ending codon preference in the chloroplast, forming a functional synergy. The optimal codons in the chloroplast genome of ‘Damianbai’ include 14 codons such as AAU (asparagine) and GAU (aspartic acid), with an ENC value of 46.1. In contrast, high-frequency codons in the mitochondria (e.g., UUU/AUU) account for 47% of the RSCU value, suggesting that mitochondria are under stronger positive selection pressure for synthesizing energy metabolism-related proteins (Yin et al., 2024). Notably, in the chloroplast genome of ‘Xilian No. 1,’ the RSCU value of AUC (isoleucine) reaches 1.98, which synergistically optimizes translational efficiency with the mitochondrial codon AUU (isoleucine) at an RSCU value of 2.83 (LIU et al., 2023). Notably, the complete absence of CUG/UUG Met codons may safeguard translation fidelity by avoiding conflicts with start codons, a feature highly conserved in cultivated tea plants (Chen et al., 2020; Li et al., 2024). This phenomenon contrasts sharply with the low retention rate of the CUG codon (<3%) in Asteraceae, suggesting that mitochondrial genomes in different groups maintain translation accuracy through distinct mechanisms (Zhang et al., 2019).

Dispersed repeats accounted for 72% of the mitochondrial genome, with the largest forward repeat measuring 8,082 bp. This abundance is comparable to that in *Camellia nitidissima* (72%) but significantly higher than in *Camellia taliensis*, suggesting that the non-homologous end-joining mechanism plays a central role in chromosome breakage and fusion (Wynn and Christensen, 2019; Li et al., 2024). In contrast to the Rosaceae mitochondrial genome, where tandem repeats account for 35%, tea plants appear to rely more on chromosome breakage/fusion events mediated by the non-homologous end-joining mechanism, rather than the typical microhomology recombination mechanism of Rosaceae (Ponty et al., 2006). The scarcity of tandem repeats (only 29) aligns with the genomic features of Rosaceae plants, indicating that tea mitochondria rely more on dispersed repeats to maintain genetic diversity rather than satellite DNA amplification (Wynn and Christensen, 2019; Li et al., 2024). Notably, in the mitochondrial genome of Asteraceae, 8–10 kb repeat sequences form sub-genomic circular structures through homologous recombination. The 8.1 kb repeat identified in this study may use a similar mechanism, suggesting that this evolutionary strategy is conserved across families in higher plants (Zhang et al., 2019). Notably, the presence of the 8.1 kb large repeat sequence may mediate sub-genomic circularization through recombination, forming a dynamic balance of multi-chromosomal structures. This mechanism is similarly validated in the mitochondrial genome of *Stewartia gemmata* (an early-branching species of *Theaceae*), where a 15.7 kb repeat sequence also drives the formation of multi-circular sub-genomes (Liu et al., 2024).

Phylogenetic analysis showed that *Camellia sinensis* cv. ‘Zhuyeqi’, *C. sinensis* (OM809792.1), and *C. sinensis* var. *assamica* (OL989850.1) formed a monophyletic group (Bootstrap = 100%), 1supporting *Camellia sinensis* cv. ‘Zhuyeqi’ as a core genotype of Chinese small-leaf tea plants (Chen et al., 2023). Its extremely low p-distance (<0.002) with the cultivated variety pubilimba reflects the influence of multi-generational artificial selection on its genome, providing molecular evidence for the “multi-origin hypothesis” of tea domestication (Chen et al., 2020; Li et al., 2024). Notably, compared to the widespread RNA editing events in 44 protein-coding genes of the *Camellia gigantocarpa* mitochondrial genome (Lu et al., 2022), the high Pi value (0.09) of key nucleo-mitochondrial interaction genes *rps4* and *atp8* in *Camellia sinensis* cv. ‘Zhuyeqi’ suggests their involvement in stress responses by regulating ribosome assembly efficiency and ATP synthesis. It also reflects convergent evolution with the positive selection signals of the mitochondrial *atp8* in sweet tea, and similar adaptive selection patterns have been observed in Solanaceae plants (e.g., potato) (Zhang et al., 2019).

The retention of 66 chloroplast homologous fragments (25,656 bp) in the mitochondria is significantly higher than that in *sweet tea* (55 fragments) and *Camellia nitidissima* (19 fragments), and far exceeds the cultivated variety *pubilimba* (40–46 fragments) and the *Assam variety* (OL989850.1, about 45 fragments) (Chen et al., 2023; Li et al., 2024). This indicates the active role of horizontal gene transfer in the evolution of tea plant organelles (Chen et al., 2023; Li et al., 2023b). The transfer of key genes such as *trnA-UGC* and *rpl2* may compensate for mitochondrial ribosome assembly defects, a mechanism showing molecular homology to the functional replacement of chloroplast *rps12* in *Arabidopsis* (Li et al., 2023b, 2024). Similarly, the transfer of *trnM-CAU* in the mitochondrial genome of *Camellia oleifera* exhibits comparable compensatory evolution (Lu et al., 2022). Notably, the retention of *trnA-UGC* may enhance translation initiation efficiency, facilitating the rapid growth of young shoots. This hypothesis aligns well with the high metabolic activity characteristic of tender leaves in cultivated tea plants (Chen et al., 2023; Li et al., 2024). A comparative analysis shows that the proportion of quadruplex repeat sequences in the mitochondrial genome of *Camellia sinensis* cv. ‘Zhuyeqi’ (43.9%) is similar to that of the Assam variety ‘Duntsa’ (43.90%) (Li et al., 2023b), but lower than that of *Camellia oleifera* (47.2%) (Lu et al., 2022). This suggests that the expansion of repeat sequences may be related to species-specific environmental adaptation.

The Ka/Ks ratios of the 37 PCGs analyzed in this study showed a wide range from 0.09 to 2.70. According to molecular evolution theory, Ka/Ks< 1 indicates purifying selection driving conservative evolution, Ka/Ks ≈ 1 reflects neutral evolution, and Ka/Ks > 1 suggests positive selection (Hurst, 2002; Lan et al., 2024). Notably, the average Ka/Ks value of the *ccmB* gene reached 1.38, significantly deviating from the neutral evolution threshold (*p*< 0.01), suggesting that this gene may have undergone adaptive evolutionary pressure. This phenomenon is similar to the positive selection pattern of the *Nad1* and *Sdh3* genes in the tea variety (*C. sinensis* var. assamica) (Li et al., 2024), but significantly differs from the overall Ka/Ks ratio (0.39) of the mitochondrial genome in *C. oleifera* (Xia et al., 2014). This indicates significant differences in evolutionary pressures on mitochondrial genes among different *Camellia* species. The high evolutionary rate of *ccmB* may be related to its functional characteristics. Similar phenomena have been reported for other metabolism-related genes, such as *atp8* (Ka/Ks = 0.81–1.65), which may indicate adaptive adjustments in energy metabolism pathways (Ye et al., 2021; Lu et al., 2023). Comparative analysis shows that the *ccmB* gene in the mitochondrial genome of *Arabidopsis thaliana* also exhibits a high Ka/Ks value (1.25-1.89) (Lu et al., 2022), suggesting that the functional plasticity of this gene in the assembly of the mitochondrial respiratory chain complex may be conserved across species. In this study, the strong conservation of *cox1* (Ka/Ks = 0.09-0.17) and *cytB* (0.21-0.47) is consistent with the conserved trend of the mitochondrial genome in *Stewartia gemmata* (Theaceae) (Liu et al., 2024), and aligns closely with the purifying selection pattern (Ka/Ks< 0.5) of complex I core genes (e.g., *nad5*) in the closely related *C. gigantocarpa* (Lu et al., 2022). This conservation may result from the functional constraints of these genes in the mitochondrial electron transport chain. However, compared to the high variability of *rps19* and *ycf1* genes in the chloroplast genome of tea plants (with a structural rearrangement frequency of 32%) (Chen et al., 2023), the structural stability of mitochondrial genes is more pronounced, indicating coordinated differences in evolutionary rates between organelle genomes. In terms of genome structure dynamics, the multi-chromosomal configuration of *Camellia sinensis* cv. ‘Zhuyeqi’ mitochondria contrasts sharply with the single circular genome (701-1,082 kb) of the tea variety Duntsa (*C. sinensis* var. *assamica* cv. Duntsa) (Li et al., 2023b). However, it is partially similar to the genome fragmentation mediated by multiple repeat sequences observed in *C. oleifera*, where the SSR density reaches 5.3/kb (Liu et al., 2024). Genome plasticity mediated by repetitive sequences, combined with functional optimization driven by natural selection, constitutes the core mechanism of organellar evolution in tea plants. However, this study has not yet addressed the co-evolutionary network between the nuclear and mitochondrial genomes. Future research should integrate high-throughput chromatin conformation capture data to reveal how nuclear-mitochondrial interactions regulate energy metabolism (Chen et al., 2020; Kong et al., 2023). At the functional evolution level, the positive selection of *ccmB* in this study complements the expansion of the acyltransferase gene family in *Camellia sinensis* var. *sinensis* (driven by whole-genome duplication events) (Wei et al., 2018), suggesting potential co-evolution between mitochondrial respiration and nuclear genome secondary metabolic pathways. In the future, a comparative analysis of the pan-mitochondrial genome map of the *Camellia* genus, combined with the purifying selection patterns (Ka/Ks< 1) of disease resistance-related genes (e.g., the *Camellia sinensis* Pectin Methylesterases family) (Huang et al., 2022), could help clarify the molecular trade-off mechanism between disease resistance and quality formation.

## 5 Conclusions

This study provides a systematic analysis of the mitochondrial genome of the tea cultivar *Camellia sinensis* cv. ‘Zhuyeqi’, revealing its multi-chromosomal structure and adaptive evolution mechanisms. The mitochondrial genome consists of seven chromosomes (total length: 911,255 bp; GC content: 45%), and its structural features are highly conserved compared to previously reported tea cultivars (GC content: 45%-46%). A total of 38 PCGs were annotated, with core functional gene clusters (e.g., *atp*, *nad*, *cox*) highly conserved among terrestrial plant mitochondria. However, the number of PCGs is significantly lower than that of the large-leaf tea cultivar *Duntsa* (47 PCGs), suggesting interspecies gene loss or annotation differences. RNA editing analysis identified 556 editing sites, with the *ccmFn* gene being a hotspot containing 38 sites, far exceeding the mitochondrial average of 139 sites in large-leaf tea cultivars. This suggests that *ccmFn* may play a unique role in mitochondrial function regulation in *Camellia sinensis* cv. ‘Zhuyeqi’. C-to-U editing dominated (98%), resulting in hydrophilicity or hydrophobicity changes in 48% of amino acids, which may mediate adaptive regulation of mitochondrial energy metabolism by altering transmembrane domain conformations.

Codon usage bias analysis showed that leucine (Leu, 10%) and tryptophan (Trp, 1%) had the highest and lowest usage frequencies, respectively. Codons with RSCU > 1 preferentially ended with A/T (AT content: 69%), reflecting co-adaptation to the high AT content of the mitochondrial genome. Repetitive sequence analysis revealed that dispersed repeats (72%) played a dominant role in genome evolution, significantly higher than that in cultivars like *Duntsa* (44%). Short repeats (<100 bp) accounted for 86%, potentially driving structural variations through homologous recombination. The phylogenetic tree clustered *Camellia sinensis* cv. ‘Zhuyeqi’ within the genus *Camellia*, showing the closest genetic distance to *Camellia sinensis* (bootstrap value: 98%). The mitochondrial genome exhibited species-specific single nucleotide polymorphisms in marker genes such as *cox1* and *nad5*, providing molecular evidence for cultivar identification. This study is the first to reveal the high density of RNA editing sites and unique repetitive sequence composition in the mitochondrial genome of *Camellia sinensis* cv. ‘Zhuyeqi’, offering critical molecular evidence for understanding the adaptive evolution of tea mitochondria. It also provides a theoretical foundation for assessing tea germplasm resources and advancing molecular breeding.

## Data Availability

The datasets presented in this study can be found in online repositories. The names of the repository/repositories and accession number(s) can be found in the article/**Supplementary Material**.
